# Georeferenced phylogenetic analysis of a global collection of wild and cultivated *Citrullus* species

**DOI:** 10.1002/ece3.7189

**Published:** 2021-01-27

**Authors:** Enoch G. Achigan‐Dako, Hervé Degbey, Iago Hale, Frank R. Blattner

**Affiliations:** ^1^ Laboratory of Genetics, Biotechnology, and Seed Science Faculty of Agronomic Sciences University of Abomey‐Calavi Cotonou Benin; ^2^ Department of Agriculture, Nutrition, and Food Systems College of Life Sciences and Agriculture University of New Hampshire Durham NH USA; ^3^ Leibniz Institute of Plant Genetics and Crop Research Gatersleben Germany

**Keywords:** biogeography, center of origin, *Citrullus* spp., colonization routes, cpDNA, Watermelon, West Africa

## Abstract

The geographical origin of watermelon (*Citrullus lanatus*) remains debated. While a first hypothesis suggests the center of origin to be West Africa, where the endemic sister species *C. mucosospermus* thrives, a second hypothesis suggests northeastern Africa where the white‐fleshed Sudanese Kordophan melon is cultivated. In this study, we infer biogeographical and haplotype genealogy for *C. lanatus, C. mucosospermus, C. amarus*, and *C. colocynthis* using noncoding cpDNA sequences (*trn*T‐*trn*L and *ndh*F*‐rpl*32 regions) from a global collection of 135 accessions. In total, we identified 38 haplotypes in *C. lanatus*, *C. mucosospermus*, *C. amarus*, and *C. colocynthis*; of these, 21 were found in Africa and 17 appear endemic to the continent. The least diverse species was *C. mucosospermus* (5 haplotypes) and the most diverse was *C. colocynthis* (16 haplotypes). Some haplotypes of *C. mucosospermus* were nearly exclusive to West Africa, and *C. lanatus* and *C. mucosospermus* shared haplotypes that were distinct from those of both *C. amarus* and *C. colocynthis*. The results support previous findings that revealed *C. mucosospermus* to be the closest relative to *C. lanatus* (including subsp. *cordophanus*). West Africa, as a center of endemism of *C. mucosospermus,* is an area of interest in the search of the origin of *C. lanatus*. This calls for further historical and phylogeographical investigations and wider collection of samples in West and northeastern Africa.

## Graphical abstract

While the origin of watermelon is still debated, two endemic close relative are separately found in northeastern Africa and West Africa. Our paper presents the current chloroplast haplotypes distribution to infer the possible colonization route of watermelon using two cultivated and two wild relatives gathered from four continents.

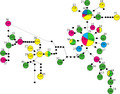

## 1 INTRODUCTION

Watermelon (*Citrullus lanatus* (Thunb.) Matsum. & Nakai) is a cultivated species of high economic importance, accounting for nearly 103.9 million metric tons of global fruit production in 2018 from 3.2 million ha (FAOSTAT, 2017). Over the last two decades, questions regarding the origin and taxonomy of *Citrullus* spp. have fuelled numerous studies to clarify phylogenetic relationships and nomenclature, identify wild relatives, and determine both centers of origin and divergence times (Chomicki & Renner, 2015; Chomicki et al., 2020; Dane et al., 2004, 2007; Dane & Liu, 2007; Dje et al., 2010; Hammer & Gladis, 2014; Jarret et al., 1997; Jarret & Newman, 2000; Levi et al., 2001, 2004, 2013; Levi & Thomas, 2005; Mujaju et al., 2013; Nesom, 2011; Renner et al., 2019; Solmaz & Sari, 2009; Solmaz et al., 2010). Despite these efforts, uncertainty vis‐à‐vis these questions remains as no wild relatives were found neither in West nor in northern East Africa; and comparatively few studies have focused on the distribution of the genetic variation within *Citrullus* or the likely colonization routes of various species within the genus.

The challenge of tracing the historical colonization routes of watermelon was for many years confounded by significant taxonomic confusion among species, subspecies, and varieties, all of which exhibit high morphological diversity. *Citrullus* Schrad. ex Eckl & Zeyh. is one of 95 genera of Cucurbitaceae (Jeffrey, 2005; Kocyan et al., 2007; Schaefer & Renner, 2011a, 2011b). To date, there seems to be a consensus regarding its complex taxonomy. According to recent research, including phylogenetic analyses and nomenclatural reviews (Chomicki et al., 2020; Renner et al., 2014) as well as a phenetic comparison within the genus (Achigan‐Dako et al., 2015), *Citrullus* encompasses the following seven species: (a) the widely cultivated *C. lanatus*, a juicy fruit found in tropical and subtropical climates including var. *cordophanus* (Ter‐Avan.) Fursa; (b) the tsamma melon *C. amarus* Schrad syn. *C. caffer* Schrad. or *C. lanatus* var. *citroides* (Bailey) Mansf., which grows in southern Africa (Whitaker & Bemis, 1976); (c) the egusi melon *C. mucosospermus* Fursa, previously referred to as a subtaxon of *C. lanatus* by many authors but which was raised to specific rank many decades ago (Fursa, 1972, 1981, 1983); (d) the bitter apple *C. colocynthis* (L.) Schrad., a perennial species growing in sandy areas throughout northern Africa and Near‐East; (e) *C. ecirrhosus* Cogn., another perennial wild species (De‐Winter, 1990); (f) *C. rehmii*, a wild annual species, with small fruits used for feeding desert animals; and (g) *C. naudinianus* (Sond.) Hook.f. from the Namib‐Kalahari region, previously placed in the genus *Acanthosicyos* Welw. ex Hook. f. and sister group to all other species. *Citrullus ecirrhosus*, *C*. *rehmii* and *C. naudinianus,* currently, are considered endemic and restricted to the desert region of Namibia with very little intraspecific variation (Dane & Lang, 2004). This understanding may however change with more extensive sampling.

Given recent clarification of *Citrullus* taxonomy, it is appropriate to revisit the question of genealogy. In a recent phylogenetic study, Chomicki and Renner (2015) indicated West Africa as the possible center of origin of *C. lanatus*, a claim at odds with earlier assertions. Indeed, whereas some experts believe watermelon originated from southern Africa, based on the distribution of wild relatives in the Namibian desert (Bates & Robinson, 1995), others point to northern or northeastern Africa, especially the Nile river area in Sudan, as the likely center of origin based on archaeological data (Paris, 2015; Renner et al., 2019; Wasylikowa & Van Der Veen, 2004). According to these latter studies, very few archaeological records of watermelon are known from southern Africa, and all date to a relatively recent period between the 8th and 13th centuries A.D. Furthermore, a cultigen is known to have been cultivated in the Nile Valley when farming was not yet practiced in southwest Africa (Zohary & Hopf, 2000). In contrast, archaeological records from West Africa are scanty, except for the presence of one endemic cultivated species (*C. mucosospermus*) previously deemed to be a subspecies or variety of *C. lanatus* (Achigan‐Dako et al., 2015; Hammer & Gladis, 2014; Nesom, 2011; Renner et al., 2014).

The fundamental questions remain: how did watermelon spread throughout the world if it has originated from West or northeastern Africa? How did the extant cultigens distribute throughout the world and how do they relate to wild types such as *C. colocynthis* or *C. amarus*? To contribute to our understanding of these questions, this paper presents a chloroplast phylogeography of *Citrullus lanatus* and three related species, one cultivated (*C. mucosospermus*) and two wild (*C. amarus* and *C. colocynthis*), using a large sample size collected from four continents. The objective is to characterize the geographical distribution of *Citrullus* haplotypes and shed specific light of the chloroplast sequence evolution *of C. lanatus*, hypothesizing that such information will help clarify our understanding of the history of this globally significant agricultural species.

## 2 MATERIALS AND METHODS

### 2.1 Taxon sampling and total genomic DNA isolation

To investigate the geographical distribution of watermelon haplotypes, we included in the study the four most economically important *Citrullus* species: (a) *C. lanatus*, widely cultivated throughout the world (78 accessions from four continents out of which only 14 were from West Africa); (b) *C. mucosospermus*, restricted to West Africa and the closest sister species of cultivated watermelon (13 accessions); (c) *C. amarus*, a wild species from southern Africa that has spread to Europe and the closest relative to *C. ecirrhosus* (22 accessions); and (d) *C. colocynthis*, a wild species found in northern Africa and East Asia (22 accessions). In total, 135 accessions were assessed, including 53 from Africa, 41 from Asia, 25 from Europe and 16 from North America (Table 1). Voucher specimens of all accessions were deposited in the herbarium of The Leibniz Institute of Plant Genetics and Crop Plant Research (IPK) (Achigan‐Dako et al., 2015).

**TABLE 1:** List of *Citrullus* accessions, their geographical origin, and accession numbers

No	Taxon	Haplotype number	Accession number	Origin	Source of collection	NBCI number for *ndh*F‐*rpl*32	NBCI number for *trn*T‐L
1	*Citrullus lanatus* var. *lanatus*	9	PI 494527	Nigeria	USDA	KX773568	KX773717
2	*Citrullus mucosospermus*	1	PI 559993	Nigeria	USDA	KX773569	KX773718
3	*Citrullus mucosospermus*	26	PI 559994	Nigeria	USDA	KX773570	KX773719
4	*Citrullus mucosospermus*	9	PI 560000	Nigeria	USDA	KX773571	KX773720
5	*Citrullus lanatus* var. *lanatus*	17	PI 560002	Nigeria	USDA	KX773572	KX773721
6	*Citrullus mucosospermus*	1	PI 560008	Nigeria	USDA	KX773573	KX773722
7	*Citrullus mucosospermus*	1	PI 560010	Nigeria	USDA	KX773574	KX773723
8	*Citrullus mucosospermus*	1	PI 560013	Nigeria	USDA	KX773575	KX773724
9	*Citrullus mucosospermus*	1	PI 560018	Nigeria	USDA	KX773576	KX773725
10	*Citrullus lanatus* var. *lanatus*	1	PI 560024	Nigeria	USDA	KX773577	KX773726
11	*Citrullus mucosospermus*	1	849 BSN 001	Benin	Prospection	KX773578	KX773727
12	*Citrullus mucosospermus*	1	975 MAT 007	Benin	Prospection	KX773579	KX773728
13	*Citrullus mucosospermus*	1	977 MAT 008	Benin	Prospection	KX773580	KX773729
14	*Citrullus mucosospermus*	1	1068 SN 045	Benin	Prospection	KX773581	KX773730
15	*Citrullus lanatus* var. *lanatus*	19	GRIF 12336	China	USDA	KX773582	KX773731
16	*Citrullus lanatus* var. *lanatus*	1	GRIF 14199	India	USDA	KX773583	KX773732
17	*Citrullus lanatus* var. *lanatus*	1	GRIF 17300	China	USDA	KX773584	KX773733
18	*Citrullus lanatus* var. *lanatus*	2	GRIF 17310	China	USDA	KX773585	KX773734
19	*Citrullus lanatus* var. *lanatus*	1	GRIF 17330	China	USDA	KX773586	KX773735
20	*Citrullus mucosospermus*	6	PI 186975	Ghana	USDA	KX773587	KX773736
21	*Citrullus lanatus* var. *lanatus*	1	PI 192937	China	USDA	KX773588	KX773737
22	*Citrullus mucosospermus*	1	PI 249010	Nigeria	USDA	KX773589	KX773738
23	*Citrullus lanatus*	1	PI 271778	South Africa	USDA	KX773590	KX773739
24	*Citrullus lanatus* var. *lanatus*	10	GRIF 55960	India	USDA	KX773591	KX773740
25	*Citrullus lanatus* var. *lanatus*	1	GRIF 55990	India	USDA	KX773592	KX773741
26	*Citrullus amarus*	3	PI 596662	South Africa	USDA	KX773593	KX773742
27	*Citrullus amarus*	4	GRIF 15896	Russia	USDA	KX773595	KX773744
28	*Citrullus amarus*	4	GRIF 15897	Russia	USDA	KX773596	KX773745
29	*Citrullus amarus*	6	PI 179881	India	USDA	KX773597	KX773746
30	*Citrullus amarus*	4	PI 189225	Democratic Republic of Congo	USDA	KX773598	KX773747
31	*Citrullus amarus*	3	PI 299378	South Africa	USDA	KX773599	KX773748
32	*Citrullus amarus*	4	PI 299379	South Africa	USDA	KX773600	KX773749
33	*Citrullus amarus*	3	PI 244018	South Africa	USDA	KX773601	KX773750
34	*Citrullus amarus*	3	PI 244019	South Africa	USDA	KX773602	KX773751
35	*Citrullus amarus*	4	PI 255137	South Africa	USDA	KX773603	KX773752
36	*Citrullus amarus*	4	PI 270563	South Africa	USDA	KX773604	KX773753
37	*Citrullus amarus*	6	PI 271779	South Africa	USDA	KX773605	KX773754
38	*Citrullus amarus*	32	PI 525,083	Egypt	USDA	KX773606	KX773755
39	*Citrullus amarus*	8	PI 596659	South Africa	USDA	KX773607	KX773756
40	*Citrullus amarus*	8	PI 596669	South Africa	USDA	KX773608	KX773757
41	*Citrullus amarus*	14	PI 596671	South Africa	USDA	KX773609	KX773758
42	*Citrullus amarus*	3	PI 596676	South Africa	USDA	KX773610	KX773759
43	*Citrullus amarus*	15	CIT 101	Ukraine	IPK	KX773611	KX773760
44	*Citrullus amarus*	4	CIT 139	Russia	IPK	KX773612	KX773761
45	*Citrullus amarus*	3	CIT 152	Zimbabwe	IPK	KX773613	KX773762
46	*Citrullus amarus*	3	CIT 310	South Africa	IPK	KX773614	KX773763
47	*Citrullus amarus*	2	CIT 313	Yemen	IPK	KX773615	KX773764
48	*Citrullus lanatus* subsp. *vulgaris*	2	CIT 207	France	IPK	KX773616	KX773765
49	*Citrullus lanatus* subsp. *vulgaris*	1	CIT 31	Ukraine	IPK	KX773617	KX773766
50	*Citrullus lanatus* subsp. *vulgaris*	1	CIT 44	Yugoslavia	IPK	KX773618	KX773767
51	*Citrullus lanatus* subsp. *vulgaris*	18	CIT 60	Croatia	IPK	KX773619	KX773768
52	*Citrullus lanatus* subsp. *vulgaris*	1	CIT 67	Italy	IPK	KX773620	KX773769
53	*Citrullus lanatus* subsp. *vulgaris*	1	CIT 69	Italy	IPK	KX773621	KX773770
54	*Citrullus lanatus* subsp. *vulgaris*	1	CIT 86	Greece	IPK	KX773623	KX773772
55	*Citrullus lanatus* subsp. *vulgaris*	1	CIT 97	Hungary	IPK	KX773624	KX773773
56	*Citrullus lanatus* subsp. *vulgaris*	1	CIT 99	China	IPK	KX773625	KX773774
57	*Citrullus lanatus* subsp. *vulgaris*	1	CIT 102	USA	IPK	KX773626	KX773775
68	*Citrullus lanatus* subsp. *vulgaris*	1	CIT 103	Russia	IPK	KX773627	KX773776
59	*Citrullus lanatus* subsp. *vulgaris*	1	CIT 105	Ukraine	IPK	KX773628	KX773777
60	*Citrullus lanatus* subsp. *Vulgaris*	1	CIT 107	Russia	IPK	KX773629	KX773778
61	*Citrullus lanatus* subsp. *Vulgaris*	1	CIT 109	Russia	IPK	KX773630	KX773779
62	*Citrullus lanatus* subsp. *vulgaris*	1	CIT 112	Ukraine	IPK	KX773631	KX773780
63	*Citrullus lanatus* subsp. *vulgaris*	2	CIT 126	Armenia	IPK	KX773634	KX773783
64	*Citrullus lanatus* subsp. *vulgaris*	1	CIT 128	Mongolia	IPK	KX773635	KX773784
65	*Citrullus lanatus* subsp. *vulgaris*	18	CIT 130	Yugoslavia	IPK	KX773636	KX773785
66	*Citrullus lanatus* subsp. *vulgaris*	1	CIT 135	Bulgaria	IPK	KX773637	KX773786
67	*Citrullus lanatus* subsp. *vulgaris*	1	CIT 142	Bulgaria	IPK	KX773638	KX773787
68	*Citrullus lanatus* subsp. *vulgaris*	1	CIT 143	Bulgaria	IPK	KX773639	KX773788
69	*Citrullus lanatus* subsp. *vulgaris*	1	CIT 156	Georgia	IPK	KX773641	KX773790
70	*Citrullus lanatus* subsp. *vulgaris*	1	CIT 158	Georgia	IPK	KX773642	KX773791
71	*Citrullus lanatus* subsp. *vulgaris*	1	CIT 160	Georgia	IPK	KX773643	KX773792
72	*Citrullus lanatus* subsp. *vulgaris*	1	CIT 164	Russia	IPK	KX773644	KX773793
73	*Citrullus lanatus* subsp. *vulgaris*	2	CIT 167	North Korea	IPK	KX773645	KX773794
74	*Citrullus lanatus* subsp. *vulgaris*	1	CIT 235	USA	IPK	KX773646	KX773795
75	*Citrullus lanatus* subsp. *vulgaris*	2	CIT 237	Japan	IPK	KX773647	KX773796
76	*Citrullus lanatus* subsp. *vulgaris*	1	CIT 239	USA	IPK	KX773648	KX773797
77	*Citrullus lanatus* subsp. *vulgaris*	1	CIT 242	USA	IPK	KX773649	KX773798
78	*Citrullus lanatus* subsp. *vulgaris*	11	CIT 244	USA	IPK	KX773650	KX773799
79	*Citrullus lanatus*	11	CIT 259	USA	IPK	KX773651	KX773800
80	*Citrullus lanatus* subsp. *vulgaris*	22	CIT 253	Japan	IPK	KX773652	KX773801
81	*Citrullus lanatus* subsp. *vulgaris*	1	CIT 303	Turkey	IPK	KX773653	KX773802
82	*Citrullus lanatus* subsp. *vulgaris*	1	CIT 306	Portugal	IPK	KX773654	KX773803
83	*Citrullus lanatus* subsp. *vulgaris*	1	06 NIA 224	Mali	Prospection	KX773656	KX773805
84	*Citrullus lanatus* subsp. *vulgaris*	2	06 NIA 567	Benin	Prospection	KX773657	KX773806
85	*Citrullus lanatus* subsp. *vulgaris*	2	07 NIA 995	Ghana	Prospection	KX773658	KX773807
86	*Citrullus lanatus* subsp. *vulgaris*	1	846 BAX1	Mali	Prospection	KX773659	KX773808
87	*Citrullus lanatus* subsp. *vulgaris*	1	1005 SE 032	Mali	Prospection	KX773660	KX773809
88	*Citrullus lanatus* subsp. *vulgaris*	1	CIT 168	North Korea	IPK	KX773661	KX773810
89	*Citrullus lanatus*	24	CIT 175	Italy	IPK	KX773662	KX773811
90	*Citrullus lanatus*	2	CIT 182	Mongolia	IPK	KX773663	KX773812
91	*Citrullus lanatus*	1	CIT 193	Ukraine	IPK	KX773665	KX773814
92	*Citrullus lanatus*	1	CIT 195	Georgia	IPK	KX773666	KX773815
93	*Citrullus lanatus*	1	CIT 200	Tajikistan	IPK	KX773668	KX773817
94	*Citrullus lanatus*	1	CIT 203	Tunisia	IPK	KX773669	KX773818
95	*Citrullus lanatus*	2	CIT 206	China	IPK	KX773670	KX773819
96	*Citrullus lanatus*	1	CIT 226	USA	IPK	KX773671	KX773820
97	*Citrullus lanatus*	1	CIT 230	Israel	IPK	KX773672	KX773821
98	*Citrullus lanatus*	1	CIT 234	USA	IPK	KX773673	KX773822
99	*Citrullus lanatus*	1	CIT 260	USA	IPK	KX773674	KX773823
100	*Citrullus lanatus*	2	CIT 264	USA	IPK	KX773675	KX773824
101	*Citrullus lanatus*	21	CIT 270	USA	IPK	KX773676	KX773825
102	*Citrullus lanatus*	1	CIT 271	Canada	IPK	KX773677	KX773826
103	*Citrullus lanatus*	1	CIT 273	USA	IPK	KX773678	KX773827
104	*Citrullus lanatus*	1	CIT 278	USA	IPK	KX773679	KX773828
105	*Citrulus lanatus* subsp. *lanatus*	16	CIT 309	South Africa	IPK	KX773680	KX773829
106	*Citrullus colocynthis*	36	CIT 150	Canary Island	IPK	KX773687	KX773836
107	*Citrullus colocynthis*	28	CIT 154	Turkmenistan	IPK	KX773688	KX773837
108	*Citrullus colocynthis*	33	CIT 166	Cape Verde	IPK	KX773689	KX773838
109	*Citrullus colocynthis*	35	CIT 190	Morocco	IPK	KX773690	KX773839
110	*Citrullus colocynthis*	12	CIT 192	India	IPK	KX773691	KX773840
111	*Citrullus colocynthis*	12	CIT 199	Egypt	IPK	KX773692	KX773841
112	*Citrullus colocynthis*	38	CIT 281	Cyprus	IPK	KX773693	KX773842
113	*Citrullus colocynthis*	13	CIT 307	Namibia	IPK	KX773694	KX773843
114	*Citrullus colocynthis*	30	PI 195927	Ethiopia	USDA	KX773695	KX773844
115	*Citrullus colocynthis*	7	PI 220778	Afghanistan	USDA	KX773696	KX773845
116	*Citrullus colocynthis*	7	PI 346082	Afghanistan	USDA	KX773697	KX773846
117	*Citrullus colocynthis*	5	PI 386014	Iran	USDA	KX773698	KX773847
118	*Citrullus colocynthis*	5	PI 386015	Iran	USDA	KX773699	KX773848
119	*Citrullus colocynthis*	5	PI 386016	Iran	USDA	KX773700	KX773849
120	*Citrullus colocynthis*	5	PI 386018	Iran	USDA	KX773701	KX773850
121	*Citrullus colocynthis*	7	PI 386021	Iran	USDA	KX773702	KX773851
122	*Citrullus colocynthis*	27	PI 386024	Iran	USDA	KX773703	KX773852
123	*Citrullus colocynthis*	29	PI 386026	Iran	USDA	KX773704	KX773853
124	*Citrullus colocynthis*	37	PI 432337	Cyprus	USDA	KX773705	KX773854
125	*Citrullus colocynthis*	34	PI 525082	Egypt	USDA	KX773706	KX773855
126	*Citrullus colocynthis*	31	PI 537277	Pakistan	USDA	KX773707	KX773856
127	*Citrullus lanatus* subsp. *vulgaris*	2	824 AE 60	Burkina Faso	Prospection	KX773708	KX773857
128	*Citrullus lanatus* subsp. *vulgaris*	23	825 AE 60	Burkina Faso	Prospection	KX773709	KX773858
129	*Citrullus lanatus* subsp. *vulgaris*	2	831 AE 031	Burkina Faso	Prospection	KX773710	KX773859
130	*Citrullus colocynthis*	25	962 KU 026	Burkina Faso	Prospection	KX773711	KX773860
131	*Citrullus lantus* cv. neri	1	06 NIA 095	Ghana	Prospection	KX773712	KX773861
132	*Citrullus lantus* cv. neri	20	06 NIA 103	Ghana	Prospection	KX773713	KX773862
133	*Citrullus lantus* cv. neri	1	06 NIA 111	Ghana	Prospection	KX773714	KX773863
134	*Citrullus lanatus vulgaris* sugar baby	2	GRIF 15895	Canada	USDA	KX773715	KX773864
135	*Citrullus lanatus vulgaris* sugar baby	2	GRIF 15898	USA	USDA	KX773716	KX773865

As indicated in Table 1, a total of 53 accessions were received from the USDA National Plant Germplasm System, 66 were received from IPK Gatersleben, and 16 were collected throughout West Africa as part of this study. Seeds of all accessions were germinated in a greenhouse at IPK‐Gatersleben, and approximately 100 mg of leaf tissue was collected from one seedling per accession and dried with silica gel. Total genomic DNA was extracted from the dried leaf tissues using the QIAGEN DNAeasy Plant Kit, and one washing step was added according to the manufacturer's instructions to increase the quality of the DNA. Concentrations were estimated on 1% agarose gels stained with ethidium bromide. Samples exhibiting suboptimal PCR amplification were purified via the QIAquick PCR Purification Kit (QIAGEN) and resuspended in 50 ml 1× TE buffer.

### 2.2 Choice of chloroplast regions

Based on the work of Shaw et al. (2007), the following nine noncoding chloroplast regions were chosen for initial screening of one accession each of *C. lanatus*, C. *mucosospermus, C. amarus* and *C. colocynthis*: *rpl*32‐*trn*L, *trn*Q‐5’*rps*16, 3’*trn*V‐*ndh*C, *ndh*F‐*rpl*32, *psb*D‐*trn*T, *psb*J‐*pet*A, 3’*rps*16‐5’*trn*K, *atp*I‐*atp*H and *trn*T‐*trn*L. For most of these regions, total levels of variation were low and exclusively interspecific. However, for *ndh*F‐*rpl*32 and *trn*T‐*trn*L, polymorphisms were observed both within and among species; thus, these two regions were selected for more in‐depth investigation. These two regions of the chloroplast genome were amplified using the following primer pairs: (a) *ndh*F (5′‐GAAAGGTATKATCAAYGMATATT‐3′) and *rpl*32‐R (5′‐CCAATATCCCTTYYTTTTCCAA‐3′); and (b) *trn*L^(UAG)^ (5′‐CTGCTTCCTAAGAGCAGCCT‐3′) and *trn*T^(GGU)^ (5′‐CCCTTTTAACTCAGTGGTAG‐3′).

### 2.3 Amplification and sequencing

PCR amplifications were performed using a Gene Amp 9700 PCR System (PE Biosystems) thermal cycler. For the *trn*T‐*trn*L region, we used a reaction volume of 50 µl consisting of 26.6 µl H_2_O, 5 µl of supply buffer (10×), an additional 2.5 µl of 15 mM MgCl_2_, 0.2 mM of each deoxynucleoside triphosphate, 10 µl Q‐solution (Qiagen), 1.5 U Taq DNA polymerase (QIAGEN), 50 pmol of each primer, and approximately 20 ng of genomic DNA. Cycling conditions for *trn*T‐*trn*L region: 95°C for 3 min; 10 cycles of 30 s at 95°C, 35 s at 56°C, and 90 s at 68°C; 35 cycles of 30 s at 95°C, 35 s at 53°C, and 90 s at 68°C; and a final extension of 10 min at 68°C. For the *ndh*F*‐rpl*32 region, PCR amplification was carried out using the Phusion Hot Start Kit (Thermo Scientific) in a reaction volume of 30 µl consisting of 17.7 µl H_2_O, 6 µl of supply buffer (10×), an additional 1.5 µl of 15 mM MgCl_2_, 0.2 mM of each deoxynucleoside triphosphate, 50 pmol of each primer, and approximately 20 ng of genomic DNA. Cycling conditions for *ndh*F*‐rpl*32 region: 98°C for 3 min; 35 cycles of 30 s at 98°C, 35 s at 58°C, and 80 s at 72°C; and a final extension of 15 min at 72°C. All PCR products were purified using the QIAquick PCR Purification Kit (QIAGEN), following manufacturer's instructions, and resuspended in 28 µl warmed 1× TE buffer. Sequencing was performed on either a MegaBACE 1000 (Amersham Biosciences) or an ABI 3730 XL (Applied Biosciences) capillary sequencer.

### 2.4 Sequence analysis and haplotype coding

For each chloroplast region, the forward and reverse sequences were manually edited and combined into a single sequence using Geneious 5.5.6 (Kearse et al., 2012). These merged sequences were submitted to NCBI GenBank to make them publicly available. Following merging, three alignments were generated: (a) species‐pairwise alignments of *C. lanatus* accessions with those of *C. mucosospermus, C. amarus*, and *C. colocynthis* for the chloroplast region *trn*T‐L; (b) the same species‐pairwise alignments for the region *ndh*F‐*rpl*32; and (c) a combined alignment of all species, containing both *trn*T‐L and *ndh*F‐*rpl*32 regions, yielding a matrix of 1,611 aligned nucleotides. In the combined alignment, for the purpose of constructing coherent and parsimonious haplotypes, repeats and indels were re‐coded as single bp polymorphisms. In the *trn*T‐L region: (a) a microsatellite ACATA at position 366 was coded as A (repeat presence) or a single gap "‐" (absence); (b) a TATT indel at position 405 was coded as a T (presence) or a single gap (absence); and (c) another TTTATA microsatellite at position 423 was coded as T (presence) or a single gap (absence). In the *ndh*F‐*rpl*32 region: (a) a poly AT, usually six to eight units (position 1149), was just replaced by a single gap for 6*(AT), A for 7*(AT), and T for 8*(AT); and (b) a TGATT microsatellite at position 1198 was coded as a T (presence) or a single gap (absence).

### 2.5 Data analysis

#### 2.5.1 Analysis of genetic diversity

Statistical parameters including sequence diversity, nucleotide diversity (Nei, 1987; Nei & Tajima, 1983), A + T content, and substitution, inversion, and transversion rates (Baier, 2011; Chiu et al., 2013; Librado & Rozas, 2009; Rozas & Rozas, 1997) were computed using DnaSP software version 5.10.01 (Chiu et al., 2013; Librado & Rozas, 2009). Pairwise intra‐ and interspecific sequence divergences for each chloroplast region were computed as the mean number of nucleotide differences per site, following the formula:

100×(Tv+Ts+ID)/L

where Tv is the number of transversions, Ts is the number of transitions, ID is the number of insertions/deletions, and L is the total length of the sequence (Dane et al., 2007; O’donnell, 1992). We used the PERMUT software package (Pons & Petit, 1996) to calculate the mean within‐population gene diversity (Ching‐Yi et al., 2012) and the total gene diversity (h_T_) (Chiu et al., 2013; Guicking et al., 2011; Martin et al., 2003; Sun et al., 2019; Zhao et al., 2019). Other intrapopulation metrics such as the number of haplotypes per population, the number of singleton haplotypes (haplotype that occurs only once in the study), the number of effective haplotypes, and the overall haplotype diversity were also estimated (Baier, 2011).

#### 2.5.2 Population differentiation and genetic structure

To infer genetic differentiation parameters, haplotypes grouped by continent or subregion were considered to comprise distinct geographic populations. We assessed the genetic differentiation among geographic populations by computing the gene differentiation statistic developed by Nei and Chesser (1983), an allele frequency‐based approach that relies on estimates of genetic differentiation among geographic subpopulations. We further used Hudson et al. (1992)’s statistical test, a simple nonparametric method based on Monte Carlos permutations. That method, compared to the traditional chi‐square analysis of genetic differentiation estimates, helped understand whether the geographical populations are genetically different from one another. In addition, genetic differentiation among populations was estimated by computing a distance matrix based on the number of mutational steps between haplotypes (Nst) and by using haplotype frequencies (Gst). Phylogeographical structure was tested based on the difference between G_ST_ and N_ST_ using PERMUT 2.0 (Chiu et al., 2013; Pons & Petit, 1996) with 1,000 permutations. In contrast to Gst, Nst considers sequence differences between the haplotypes. Thus, Nst > Gst indicates that closely related haplotypes are observed more often in a given geographical area than would be expected by chance (Burban et al., 1999; Chávez‐Pesqueira & Núñez‐Farfán, 2016; Chiu et al., 2013; Grivet, 2002; Guicking et al., 2011; Pons & Petit, 1996; Sun et al., 2019). Following Templeton (1996), we tested the null hypothesis of homogeneity of nucleotide mutations using Fisher's exact test to investigate haplotypic differentiation within the overall population. We also performed Fu's *Fs* (Fu, 1997) to analyze the expansion level of the population under the hypothesis of selective neutrality and population equilibrium. Tajima's D test also was implemented for comparison with the Fu's *Fs*.

#### 2.5.3 Statistical parsimony network

Parsimony networks were constructed to infer phylogeographical relationships among haplotypes using TCS v1.21 (Clément et al., 2000). TCS estimates genealogical relationships of sequences and collapses identical sequences into haplotypes (HT). To account for the different mutation rates underlying base substitutions, indels, and microsatellites, we followed the two‐step strategy described by Bänfer et al. (2006) and performed by Guicking et al. (2011). The network was re‐drawn from the TCS output using Adobe Illustrator.

## 3 RESULTS

### 3.1 Nucleotide variations, intra‐ and interspecific diversity

The length of the amplified *trn*T‐*trn*L region within *C*. *lanatus* ranged from 951 to 954 bp. No parsimony‐informative site was found within *C. lanatus*, but 3 indels were found at positions 242, 295, and 296. The amplified *ndh*F‐*rpl*32 region ranged from 650 to 652 bp in the species, also with no parsimony‐informative site, though 5 indels were found at positions 970, 1,028, 1,143, 1,178, and 1,198 (Table S1). The combined length of the two cpDNA regions was found equal to 1,601–1,605 bp and included 1 SNP (position 1,399) and 1 microsatellite (position 366); but no polymorphisms were parsimony‐informative. In total, the sampled accessions of this species comprise 12 distinct haplotypes, among which 10 were singletons, with an overall haplotype diversity of 0.5656 (Table 2).

**TABLE 2:** Genetic statistics based on the *trn*T‐L, *ndh*F‐*rpl*32, and their combination in *Citrullus* spp

CpDNA regions	Taxonomic groups	Number of accessions	Total Length (bp)	Parsimony‐informative sites	Number of haplotypes	Haplotypes diversity	Nucleotide diversity (Pi)	Average number of nucleotide difference (k)	Indel events	A + T (%)
*trn*T‐L	*Citrullus lanatus*	78	951–954	0	4	0.44	0	0	3	76.1
	*C. mucosospermus*	16	950–953	0	3	0.34	0	0	2	75.8
	*C. amarus*	22	950–953	0	5	0.52	1 × 10^–4^	0.09	4	75.9
	*C. colocynthis*	22	948–954	6	12	0.92	28 × 10^–4^	2.65	5	76.0
*ndh*F‐*rpl*32	*C. lanatus*	78	650–652	0	8	0.24	0.4 × 10^–4^	0.027	5	76.3
	*C. mucosospermus*	16	651–652	0	3	0.25	1.9 × 10^–4^	0.125	0	76.8
	*C. amarus*	22	651–653	2	6	0.71	10.5 × 10^–4^	0.68	1	76.8
	*C. colocynthis*	22	650–653	1	11	0.80	7 × 10^–4^	0.45	6	76.3
*trn*T‐L&*ndh*F‐*rpl*32	*C. lanatus*	78	1601–1605	0	12	0.56	0.2 × 10–4	0.025	8	76.2
	*C. mucosospermus*	16	1601–1604	0	5	0.53	0.8 × 10–4	0.125	2	76.2
	*C. amarus*	22	1602–1604	2	8	0.81	4 0.8 × 10–4	0.78	6	76.2
	*C. colocynthis*	22	1599–1605	7	16	0.96	19.5 × 10–4	3.10	12	76.1

Note

Parsimony‐informative sites: Polymorphic sites with a minimum of two alleles that are each present at least twice in the population.

Noninformative sites: Polymorphic sites that are unique in the population (singleton sites).

Haplotype diversity: The probability that two given sequences from two different haplotypes belong to two different regions or populations.

Nucleotide diversity: The average number of nucleotide substitutions per site between two sequences (Lynch and Crease 1990).

Average number of nucleotide differences: The average number of nucleotide differences (either Indels or SNPs) within a given population.

Indel events: The number of insertions/deletions in the genomic region.

A + T (%): A + T content in the genomic region.

Sequence lengths within *C. mucosospermus* were similar, with the combined length of the two regions spanning by 1,601–1,604 bp. One SNP (nonparsimony informative) was identified in the *ndh*F‐*rpl*32 region (position 1,397), as well as two indels in *trn*T‐*trn*L region (positions 242 and 296). Of the five haplotypes found among the sampled accessions of this species, three were singletons; and overall haplotype diversity is 0.5333.

The combined sequence length in *C. amarus* ranged between 1,602–1,604 bp (950–953 bp in *trn*T‐*trn*L and 651–653 bp in *ndh*F‐*rpl*32) and contained ten polymorphic sites. Of those, 4 indels were observed in *trn*T‐L (positions 295, 296, 297, 405) and 1 in *ndh*F‐*rpl*32 (positions 1,198). Four SNPs were found at positions 918, 1,149, 1,397, and 1,526; and there is a microsatellite at position 1,149. *C. amarus* was characterized by eight haplotypes, among which six were private; and overall haplotype diversity is 0.81.

*Citrullus colocynthis* was characterized by a combined sequence length of 1,599–1,605 bp (948–954 bp for *trn*T‐*trn*L and 650–653 bp for *ndh*F‐*rpl*32) that features 10 SNPs (positions 406, 455, 487, 882, 918, 949, 1,111, 1,286, 1,397, and 1,526) and 3 microsatellites (positions 366, 423, 1,149). In addition, there were 11 indels (positions 199, 242, 295, 296, 297, 972, 1,179, 1,180, 1,200, 1,262, and 1,530), 7 of which were parsimony informative (6 within *trn*T‐*trn*L and 1 within *ndh*F‐*rpl*32). The collection of this species contains 16 haplotypes, all private, and has an overall haplotype diversity of 0.96.

**FIGURE 1:**
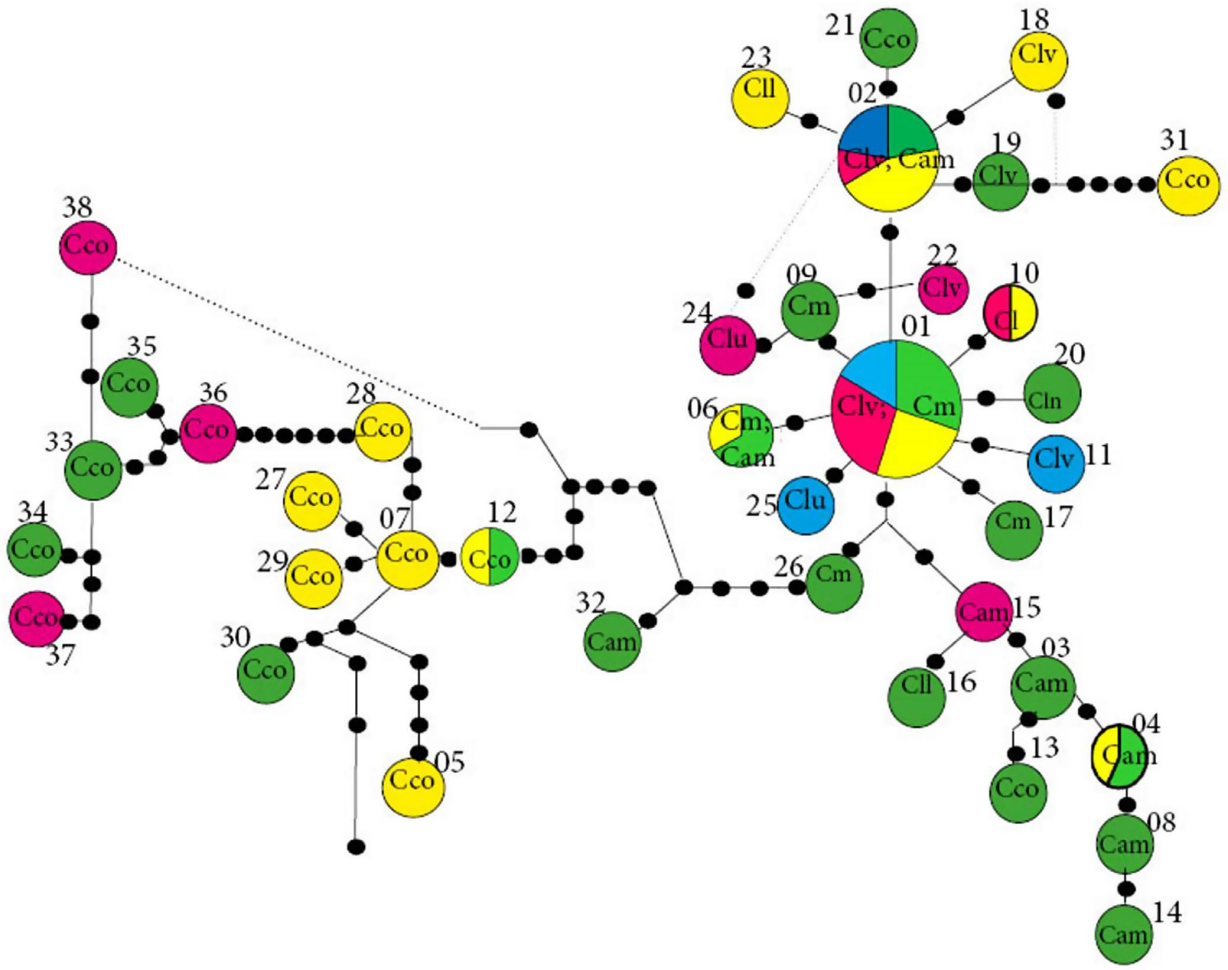
TCS network of 38 *Citrullus* spp. haplotypes. Circle size is proportional to haplotype frequency. Taxon names are abbreviated with two or three letters. Clv: *C. lanatus* subsp. *vulgaris*; Cll: *C. lanatus* subsp. *lanatus*; Cm: *C. mucosospermus*; Cam: *C. amarus*; and Cco: *C. colocynthis*. The numbers are arbitrary haplotype ID numbers (see Table S1), and the colors indicate geographical distribution: Africa (green), Asia (yellow); Europe (red), and North America (blue)

Based on the 29 polymorphic sites detected within the two cpDNA regions, 38 haplotypes were detected among the sampled accessions (Table 3). The most ancient haplotype (H1), according to TCS analysis, is exclusive to the cultivated species *C. lanatus* and *C. mucosospermus*. Of the 26 singleton haplotypes detected, 13 (50%) were found within *C. colocynthis*, indicating recent haplotype divergence in that species (Figure 1).

**TABLE 3:**
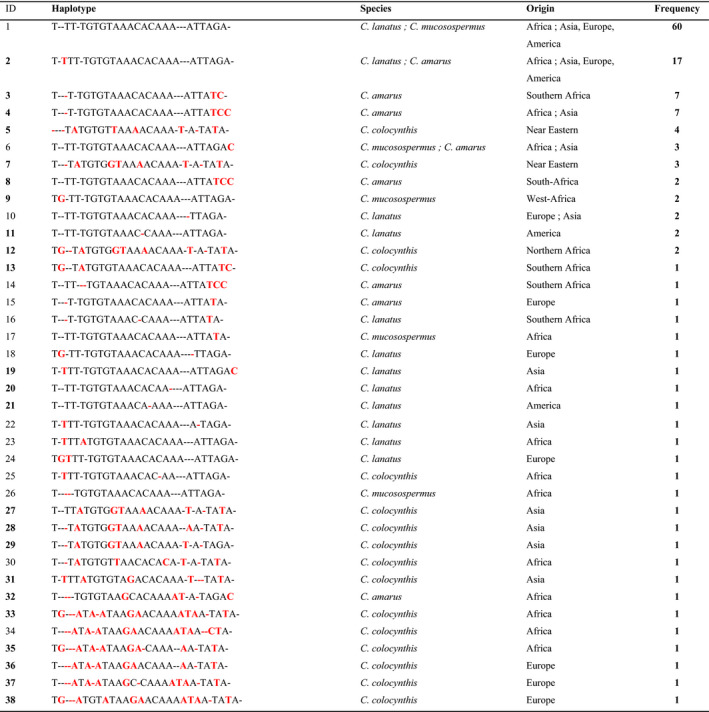
Haplotype codes for the combined *trn*T‐L and *ndh*F‐*rpl*32 chloroplast regions for the global collections of the four *Citrullus* species in this study

Note

Red colour letters highlight sequence variations

### 3.2 Geographical distribution, genetic differentiation of haplotypes, and population expansion

The pattern of polymorphism suggested non neutral selection as revealed by both Fu's *Fs* statistic and Tajima's *D* (*F*s = −3.624, *p* = 0.016; D: −0.59858; not statistical significant, *p* > 0.10). Moreover, Ficher's exact test used to investigate haplotypic differentiation within the overall population suggested the rejection of the null hypothesis of homogeneity of nucleotide substitutions (LD = 0.1958, *p* < 0.001) following the neutral theory of molecular evolution.

**FIGURE 2:**
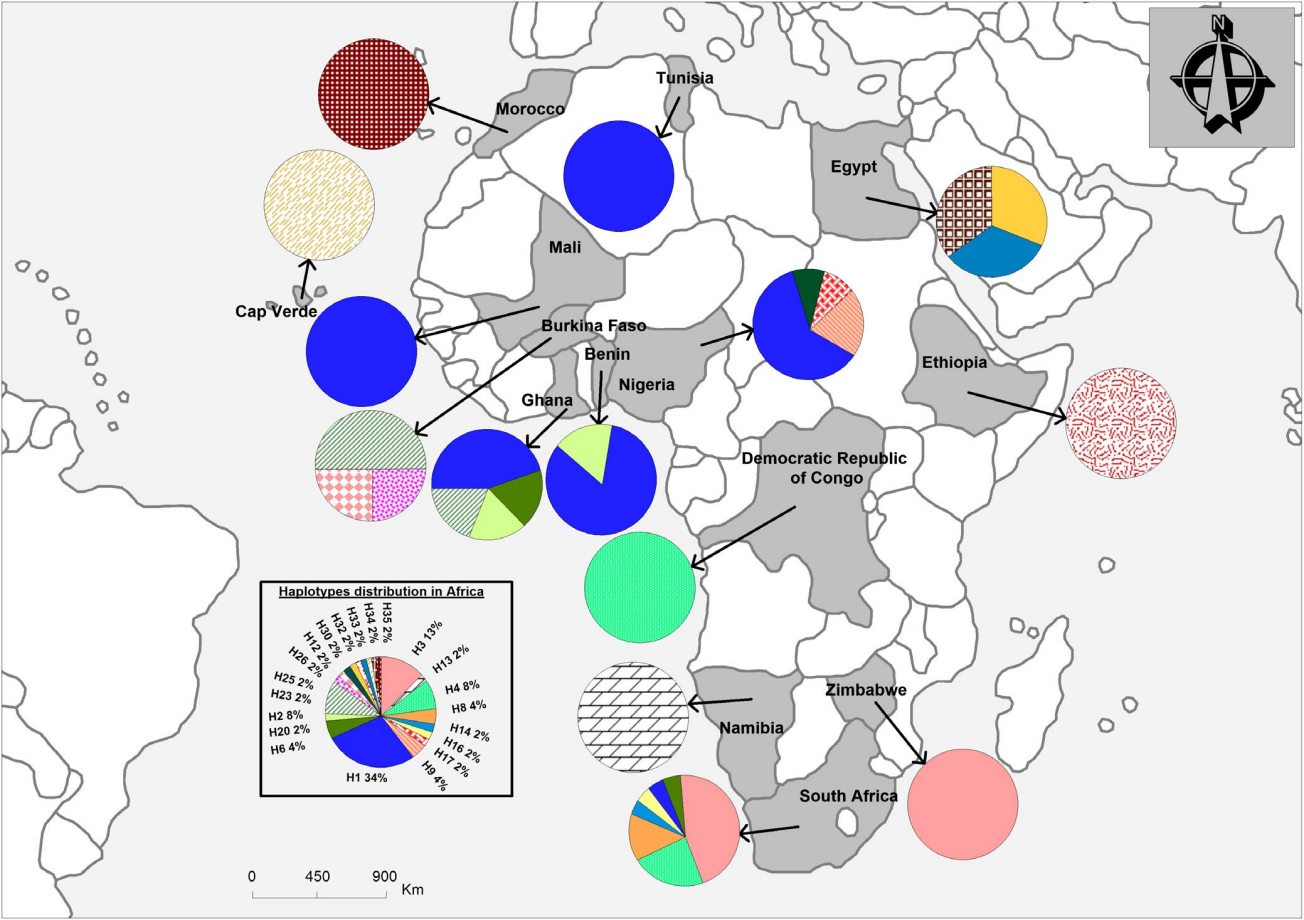
Distribution and frequencies of *Citrullus* spp. haplotypes in Africa

**FIGURE 3:**
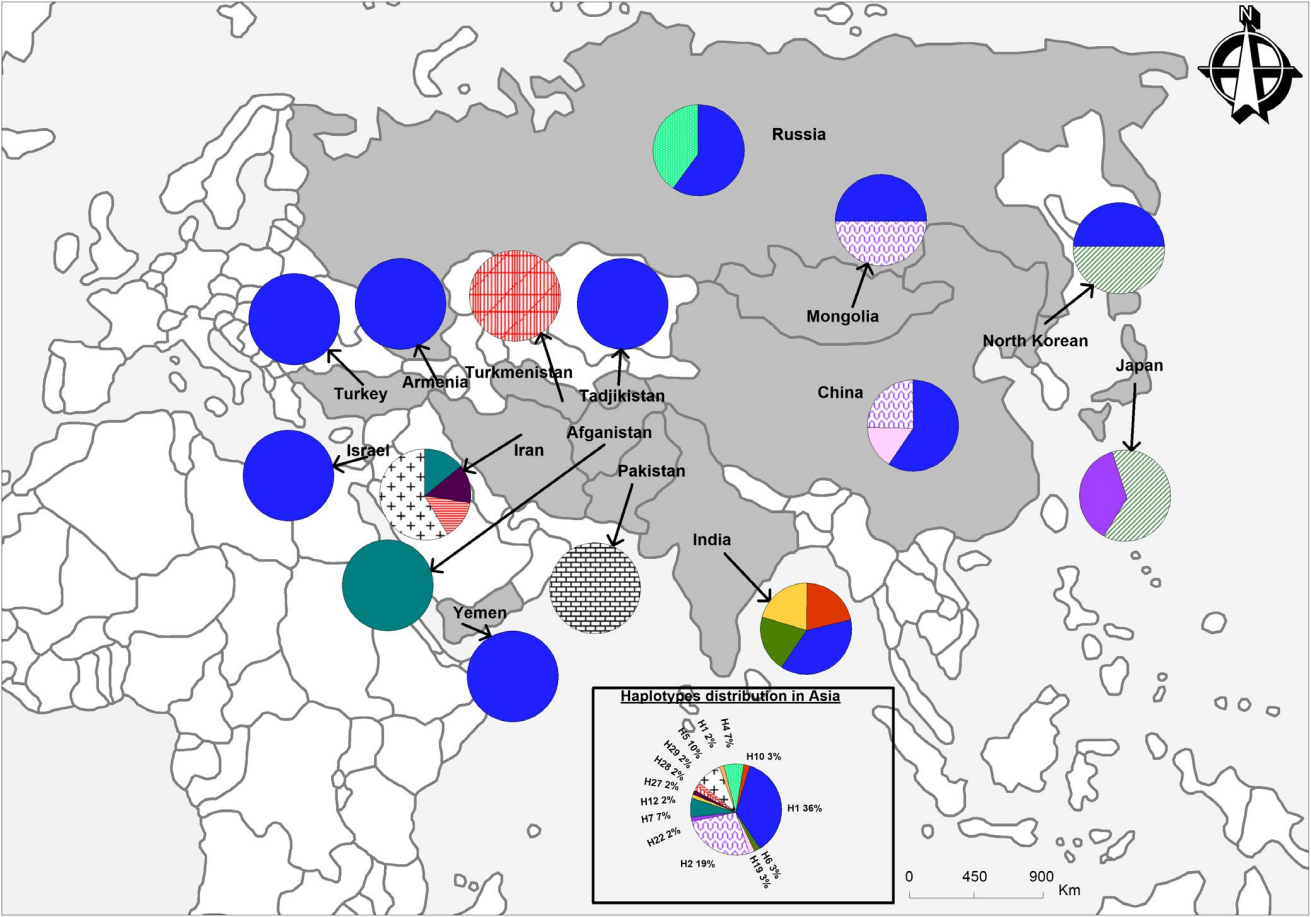
Distribution and frequencies of *Citrullus* spp. haplotypes in Asia

**FIGURE 4:**
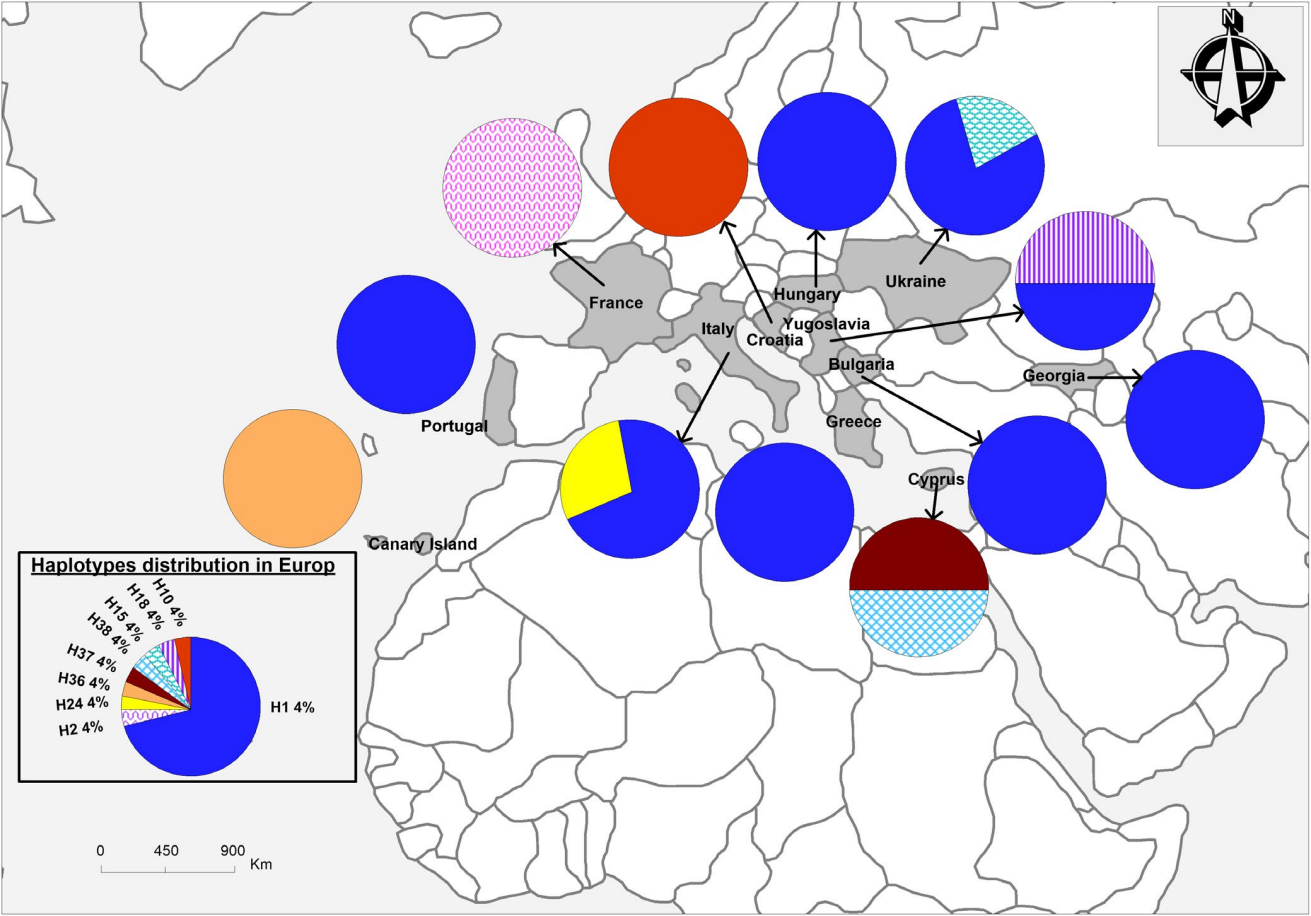
Distribution and frequencies of *Citrullus* spp. haplotypes in Europe

**FIGURE 5:**
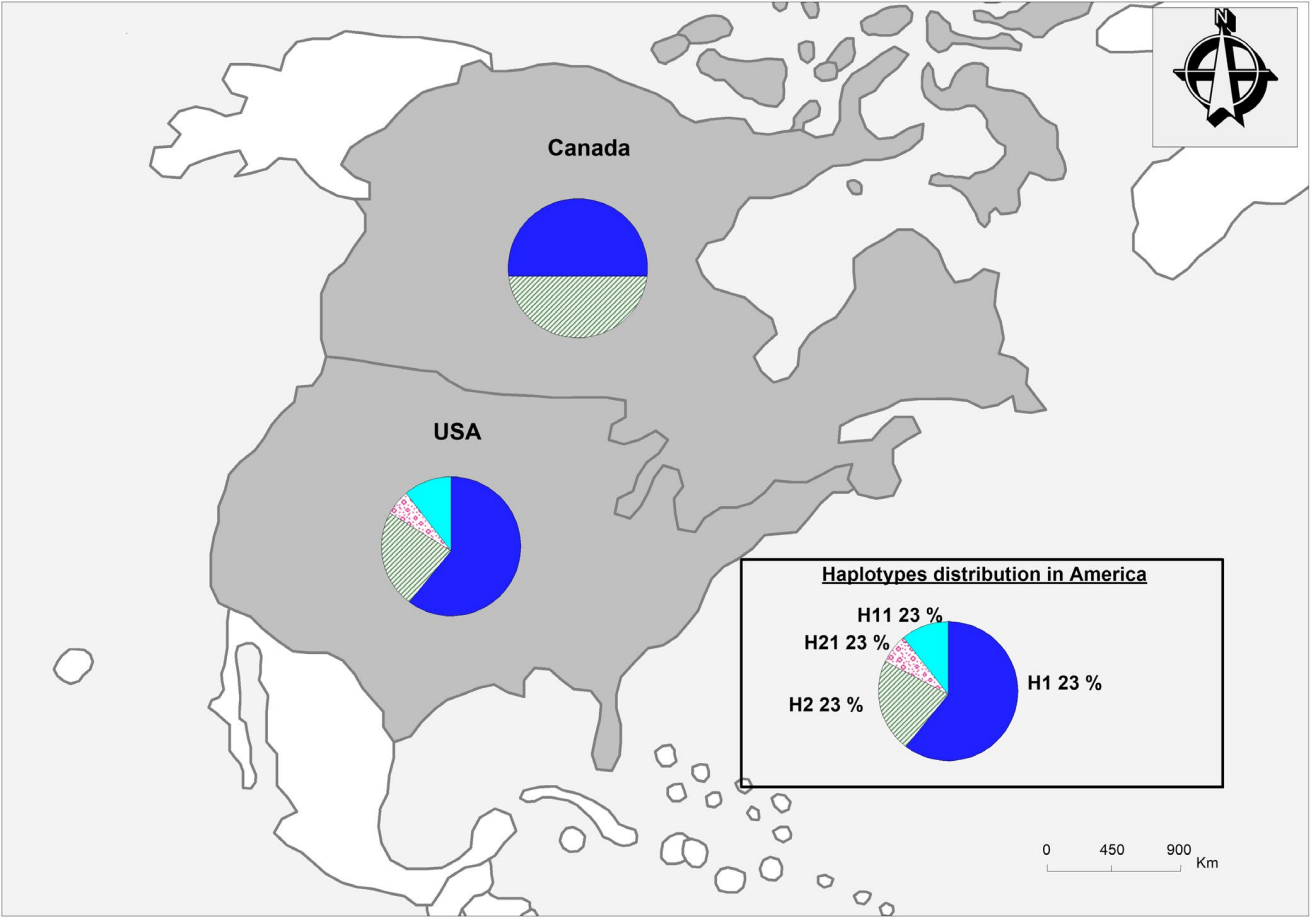
Distribution and frequencies of *Citrullus* spp. haplotypes in North America

Within‐continent gene diversity (Hs) varied from 0.57 (in Europe) to 0.85 (in Africa), with the majority of haplotypes being specific to certain regions. For instance, of the 21 haplotypes found in Africa, 16 were specific to the continent; of the 14 haplotypes found in Asia, eight were specific; of the nine found in Europe, six were specific; and of the four recovered from America, two were specific to that region (see Figures 2, 3, 4, 5).

Haplotypes of *C. mucosospermus* were almost uniquely restricted to West Africa, and *C. amarus* haplotypes appeared specific to southern Africa. Haplotypes of *C. colocynthis* shared by Namibia, Ethiopia, and northern Africa were also found widespread throughout Asia. Across that continent, some haplotypes of *C. colocynthis* were specific to different countries (Figure 1). Six *C. colocynthis* haplotypes were specific to Asia, and six were specific to Africa. For this species, Iran contributed the highest number of haplotypes in Asia (Figure 1), as Egypt did in Africa (Figure 1).

Within *C. lanatus*, although all regions shared most haplotypes, Africa exhibited the highest number of singletons. The ancient haplotype H1 was found not only among West African countries but also in Europe (Georgia, former Yugoslavia, Italy, and Ukraine), Asia (Russia, Japan, China, India), and North America (United States and Canada). North Africa (Egypt) and southern Asia (India) shared *C. colocynthis* haplotype H12; and haplotype H4, specific to *C. amarus*, was shared by African countries (e.g., South Africa and the Democratic Republic of Congo) and Russia (Figure 1). Haplotype H2 was found throughout West Africa (Benin, Burkina‐Faso, and Ghana) as well as in Asia (China, Japan, Yemen, North‐Korean Republic, Mongolia, and Armenia), France, and North America (United States and Canada). Haplotype H2 is shared by *C. lanatus* and *C. amarus*; and haplotype H6 is shared by *C. mucosospermus* and *C. amarus* species (see Figures 2, 3, 4, 5).

Analysis of interspecific genetic differentiation revealed a high level of total genetic differentiation among continents (Tables 4 and 5). Coefficients of pairwise genetic differentiation values were highest between Africa and Europe, on the one hand, and Asia and Europe, on the other; Gst was lower between Africa and Asia (0.006). The coefficient of population differentiation Gst was 0.196, and the pairwise difference between haplotypes Nst = 0.374.

**TABLE 4:** Diversity and differentiation statistics for the four Citrullus spp. in this study, based on combined cpDNA haplotypes, according to Pons and Petit (1996) and adapted from Guicking et al. (2011)

Genetic parameters	Value	Standard error
Expected mean within‐population gene diversity (h_S_)	0.737	0.0671
Expected total gene diversity (h_T_)	0.917	0.0320
Expected coefficient of genetic differentiation (Gst)	**0.196**	0.0812
Observed mean within‐population gene diversity (Vs)	0.668	0.1878
Observed total gene diversity, accounting for similarities among haplotypes (V_T_)	1.067	0.1609
Observed coefficient of genetic differentiation (Nst)	**0.374**	0.1274

Note

h_S_: The average permuted value of gene diversity within the four geographical regions (Africa, America, Asia, and Europe).

h_T_: The permuted value of gene diversity across all four geographical regions.

G_St_: The permuted value of genetic differentiation among the four geographical regions.

V_S_: The average observed value of gene diversity within the four geographical regions.

V_T_: The observed value of gene diversity across all four geographical regions.

N_St_: The observed value of genetic differentiation among the four geographical regions.

**TABLE 5:** Pairwise genetic differentiation between continents (a), between African regions (b) and between Asian regions (c)

Region 1	Region 2	Hs	Ks	Kxy	Gst	Chi‐square
a: Pairwise genetic differentiation between continents (Hudson, 1992)						
Africa	Asia	0.85	0.85	4.78	0.006	χ^2^ = 135.067 *p*‐value = 0.05
Africa	Europe	0.76	0.76	3.84	0.035	
Africa	America	0.81	0.81	2.92	0.023	
Asia	Europe	0.73	0.73	4.41	0.038	
Asia	America	0.77	0.77	3.43	0.014	
Europe	America	0.57	0.57	2.12	0.0079	
b: Pairwise genetic differentiation between African regions (Hudson, 1992)						
West Africa	South Africa	0.73	1.92	3.79	0.12	χ^2^ = 84.02 *p*‐value = 0.0001
West Africa	South Africa	0.72	3.14	9.02	0.043	
South Africa	North Africa	0.85	3.88	9.34	0.05	
c: Pairwise genetic differentiation between Asian regions (Hudson, 1992)						
East Asia	West Asia	0.77	3.50	6.30	0.04	χ^2^ = 65.75 *p*‐value = 0.0047
East Asia	South Asia	0.76	2.65	4.73	0.06	
East Asia	North Asia	0.64	1.30	2.37	0.09	
West Asia	South Asia	0.89	6.20	6.20	0.014	
West Asia	North Asia	0.78	4.97	6.64	0.08	
South Asia	North Asia	0.77	4.19	5.11	0.07	

Note

Hs: The mean within‐continent gene diversity.

Ks: A weighted average of the number of differences between sequences from continents *i* and *j*.

Kxy: The average number of differences between two samples, regardless of their provenance.

GST: The coefficient of genetic differentiation between continents.

## 4 DISCUSSION

### 4.1 Genetic diversity and sequence variation

Within the genus *Citrullus* genetic diversity analyses have been conducted since the second half of the 20th century (Hashizume et al. 1996) revealing various trends. Previous knowledge revealed lower genetic diversity in *Citrullus* for breeding purpose (Levi et al., 2001, 2004). Recent studies shed light on obvious genetic diversity within the genus. For instance, a study using High Frequency Oligonucleotide Target Active Genes (HFO‐TAGs) revealed high genetic diversity among *Citrullus* spp. and highlighted the potential importance of PI accessions as sources of valuable traits like disease resistance (Levi et al., 2013).

Our findings revealed low cpDNA variability among *C. lanatus* and *C. mucosospermus*. This was also observed by Dane and Lang (2004) and Dane et al. (2004) who found low nucleotide variability based on a low number of parsimony‐informative sites within each of the studied species. Most haplotypes were found within noncultivated (*C. colocynthis*) rather than cultivated (*C. lanatus* and *C. mucosospermus*) species. Taxa were clearly separated from one another with divergence based mainly on indels and transition events (Dane et al., 2004). However, there was sufficient resolution of the *trn*T‐L and *ndh*F‐*rpl*32 noncoding regions to reveal intraspecific variability.

Chloroplast sequence analysis revealed that the *ndh*F‐*rpl*32 region exhibits comparatively higher variability within the two cultivated species than the *trn*T‐L region. Dane and Lang (2004) analyzed four chloroplast regions (*nhd*F, *ycf*6‐*psb*M, *ycf*9‐*trn*G and *atp*A‐*trn*R) and found no variability within cultivated accessions, grouped either by morphological traits or geographical origin. In this study, we used a large number of *C. lanatus* accessions from a wide geographical range and observed low haplotype diversity within that species, as also revealed by Guo et al. (2013). While many factors can influence sequence diversity, selection is a major contributor via the imposition of bottlenecks that can substantially reduce diversity (Dane & Lang, 2004; Levi et al., 2013). The lack of haplotype divergence within *C. lanatus* and *C. mucosospermus* is likely the result of selection or other bottlenecks in the domestication histories of watermelon and egusi melon. Certainly, selection for sweet red‐fleshed cultivars with high lycopene content or selection of seed type as source of protein/oil for consumption might contribute to current genetic structure in those cultivated species (Achigan‐Dako et al., 2015; Renner et al., 2019).

*Citrullus colocynthis* exhibited a relatively high number of parsimony‐informative characters. Dane et al. (2004) revealed that haplotypes detected within *C. colocynthis* were associated with geographical origin and that was also confirmed by Levi et al. (2017). The haplotype diversity within *C. colocynthis* suggests cryptic evolution and calls for a comprehensive morphological comparison of Asian and African colocynths. Such an investigation is exemplified by the recent studies on *Cucumis melo* that revealed modern melon cultivars go back to two lineages and was domesticated at least twice: in Asia and in Africa (Endl et al., 2018).

### 4.2 Citrullus haplotype evolution

Thirty‐eight haplotypes were detected among the cultivated and wild *Citrullus* accessions used in this study. Dane et al. (2004) found seven haplotypes within the genus, using 55 accessions of *C. lanatus,* 15 accessions of *C. colocynthis*, and a total of seven cpDNA regions. With two cpDNA regions and 135 accessions carefully selected to represent a wide geographical region, we detected an even higher haplotype diversity among *Citrullus* spp. This situation can be expected to continue to evolve as more watermelon accessions from Sudan or northeast Africa are sequenced, particularly, the Sudanese sweet white‐fleshed melon. Unfortunately, sampling of *C. lanatus* from the Darfur region of Sudan has been scarce (Renner et al., 2019).

On average, we observed 9.5 haplotypes per species, varying from 5 to 16. In comparison with other species, Guicking et al. (2011) found 9.8 haplotypes per species in *Macaranga* and Jakob and Blattner (2006) found 2.83 haplotypes per species in *Hordeum*. In *Citrullus* spp., nucleotide substitutions appear to have evolved at different rates, an observation supported by the Fisher's test for homogeneity of nucleotide substitution. Fu's test *Fs* also rejected the null hypothesis of neutrality of evolution of nucleotide substitution, further supporting the hypothesis that the polymorphism pattern observed is nonrandom. Population expansions tend to produce significantly negative values of D, while population bottlenecks tend to produce significantly positive values of D. In our case the departure from neutrality might indicate that there is a high demographic expansion and a pattern of isolation by distance would be occurred between the continents (Jiang et al., 2016).

### 4.3 Genetic differentiation and geographical structure

The coefficient of population differentiation (Gst), that uses allelic frequencies and does not take into account the distances among haplotypes, and the coefficient of differentiation (Nst) based on the pairwise difference between alleles were found respectively, equal to 0.196 and 0.374; but the difference was not significant (*p* > 0.05). In *Citrullus* spp. Mujaju et al. (2011) found Gst = 0.56 and Nst = 0.49 for sweet watermelon and Gst = 0.71, Nst = 0.81 for cow watermelon. The fact that the differentiation parameter based on the pairwise difference between alleles is greater than the one calculated without permutation (i.e., Nst > Gst) indicates that the collection is characterized by clear geographic structure (Dane et al., 2007; Grivet, 2002; Guicking et al., 2011). Also, the significant value of the total gene diversity across all four geographical regions (hT = 0.917, standard error = 0.0320) is indicating a strong structure in the population (Pons & Petit, 1996; Sun et al., 2019; Zhao et al., 2019).

Levi et al. (2017) observed that accessions of *C. colocynthis* were subdivided into five groups in general agreement with their centers of diversification and origin. Our findings indicated that regional genetic differentiation statistics support Levi et al. (2017)’s conclusions, with subsamples from different regions exhibiting genetic differentiation associated with their likely centers of diversification. Also, haplotypes of *C. amarus* were mostly grouped in Southern Africa, which is assumed to be the origin of that species (Chomicki & Renner, 2015; Dane & Liu, 2007).

*Citrullus* chloroplast sequences analysis with TCS 1.21 resulted in a network where haplotypes widely sampled throughout West Africa were placed at the root. While coalescence theory predicts that older alleles will prevail in a population due to a higher number of descending lineages and associated wider geographic distributions (Crandall & Templeton, 1993), such an observation may depend on sample sizes and evolutionary/domestication histories and also the lack of subsp. *cordophanus* (from northeast Africa) in the germplasm studied. In this study, H1 is the most frequently sampled haplotype and has the most connections with other haplotypes; thus, H1 may be considered the most ancient haplotype. This ancient haplotype was sampled most frequently in West Africa (i.e., Nigeria and Benin) and was highly shared by accessions of both *C. lanatus* and *C. mucosospermus*. These results support the findings of Chomicki and Renner (2015) and Renner et al. (2019) who used eleven gene regions to infer phylogeny of *Citrullus* species, and also a 3,500‐year‐old leaf sample from the Egyptian tomb to infer close relationship between *C. lanatus* and *C. mucosospermus*. Our findings, based upon a large set of egusi melon and watermelon accessions from four continents, provide further evidence of that close relationship between these two species. However, they are indeed two different species, as previous crosses between them (e.g., Charleston Gray x PI 560006) resulted in high levels of sterility (Gusmini et al., 2004). The very limited haplotype diversity among the two species suggests an old split with chlorotype fixation (Dane & Liu, 2007) and ancient types of *C*. *mucosospermus* originating from West Africa (Renner et al., 2014). However, to the best of our knowledge, no wild populations have been confirmed in West Africa. Spontaneous plants may have been found earlier, but those individuals certainly escaped from cultivation. A region‐wide collecting mission by the first author yielded no wild population of *C. mucosospermus* in West Africa (Achigan‐Dako et al., 2015) though, the presence in West Africa of the “neri” type [figure 9f in Achigan‐Dako et al. (2015) and figure 1 in Minsart et al. (2011)], another cultivated egusi melon that exhibits smaller seeds with yellow soft coat, should be highlighted as a contributor to the genepool of *Citrullus* is the region. While this neri type (*C. lanatus*) is morphologically distinct from *C. mucosospermus*, it has been rarely studied.

Archaeological evidence indicates the northeast of Africa as a center of origin and domestication (Chomicki et al., 2020). Authors reported wild dessert watermelon in that region (Paris, 2015) or the genetic affinity with the *C. lanatus* var. *cordophanus* (a sweet white‐fleshed cultivar) (Renner et al., 2019). However, within the genus *Citrullus mucosospermus* remains the closest relative species to *C. lanatus*. The presence of an ancient haplotype in West Africa on the one hand and the close relationship between *C. lanatus* and subsp. *cordophanus* of Darfur in northeastern Africa as revealed by Renner et al. (2019) on the second hand, calls for further molecular and archaeological investigations to generate sufficient knowledge on newly published results, including those reported here. New molecular investigations should include more materials from Sudan and neighboring countries where wild populations of watermelon have been found (Paris, 2015). Moreover, our data showed that one of the Egyptian accessions (PI 525083), indicated to be *C. amarus* and observed by Levi et al. (2013) to cluster with dessert watermelon, exhibits a unique haplotype (H32). That accession is several mutations away from *C. colocynthis* and closer to watermelon and egusi melon haplotype. Previous findings of Levi et al. (2017) showed that PI 525083 rather clustered with *C. lanatus* var. *lanatus*. In addition, the hypothesis that watermelon is from northeastern Africa does not explain how an endemic species such as *C. mucosospermus* shares the same haplotype with dessert watermelon, while other accessions from the region (e.g., PI 525083) shows unique haplotype. If *C. lanatus* did indeed spread to the world from West or northeastern Africa, how and when was it domesticated in those regions as New Kingdom Egyptians were cultivating sweet red‐fleshed watermelon more than 3,500 years ago? From which species was *C. mucosospermus* domesticated? Through what mechanisms was *C. lanatus* spread to Asia and when? More germplasm collections from all continents are necessary to fully understand the phylogeographical relationships among *Citrullus* species. In Africa, the focus should be on both west and northeastern regions to resolve the domestication history of modern cultivars.

## 5 CONCLUSION

The genus *Citrullus* includes seven species that may originate from different parts of the world, according to previous and current data. Our results reveal 38 distinct chloroplast haplotypes among *Citrullus* spp. and the distribution of those haplotypes across the world. The close relationship of egusi melon and Kordofan melon to watermelon raised new questions regarding the colonization routes of major crops and the current status of extant genetic diversity of wild relatives in places of origin.

## CONFLICT OF INTEREST

The authors declare that they have no conflict of interest.

## AUTHOR CONTRIBUTIONS

**Enoch G. Achigan‐Dako**: Conceptualization (equal); Data curation (equal), Formal analysis (equal); Funding acquisition, Investigation (equal); Methodology (equal); Project administration (equal); Supervision (equal); Visualization (equal); Writing‐original draft (equal); Writing‐review and editing (equal). **Hervé Degbey**: Data curation (equal), Formal analysis (equal); Investigation (equal); Methodology (equal); Visualization (equal); Writing‐original draft (equal); Writing‐review and editing (equal). **Iago Hale**: Methodology (equal); Visualization (equal); Writing‐original draft (equal); Writing‐review and editing (equal). **Frank Blattner**: Conceptualization (equal); Investigation (equal); Methodology (equal); Project administration (equal); Supervision (equal); Visualization (equal); Writing‐review and editing (equal).

## Supporting information

Table S1

## Data Availability

DNA sequences: NCBI GenBank accession numbers are provided in Table S1. Dryad: https://doi.org/10.5061/dryad.31zcrjdjw
