# Supplementary material for: Georeferenced phylogenetic analysis of a global collection of wild and cultivated Citrullus species
Source: Ecol Evol. 2021 Jan 27;11(4):1918–36. doi: 10.1002/ece3.7189 (PMC7882934; doi:10.1002/ece3.7189)
Supplement: Supplementary file 1 — Table S1 [file ECE3-11-1918-s001.docx]

**Table S1.** Polymorphisms and haplotype variants observed in two chloroplast regions of *Citrullus* spp. (i: indels, m: microsatellites, s: SNPs, Var: variant)

| **Polymorphism** | **Region** | **Position** | **Type** | **Var1** | **Var2** | **Var3** | **Variant haplotypes codes** | | | | | | | | | | | | | | | | | | |
| --- | --- | --- | --- | --- | --- | --- | --- | --- | --- | --- | --- | --- | --- | --- | --- | --- | --- | --- | --- | --- | --- | --- | --- | --- | --- |
|  |  |  |  |  |  |  | **1** | **2** | **3** | 4 | **5** | **6** | **7** | **8** | **9** | **10** | **11** | **12** | **13** | **14** | **15** | **16** | **17** | **18** | **19** |
| 1 | *trn*T_L | 199 | i | T | - |  | T | T | T | T | - | T | T | T | T | T | T | T | T | T | T | T | T | T | T |
| 2 | *trn*T_L | 242 | i | G | - |  | - | - | - | - | - | - | - | - | G | - | - | G | G | - | - | - | - | G | - |
| 3 | *trn*T_L | 295 | i | T | - |  | - | T | - | - | - | - | - | - | - | - | - | - | - | - | - | - | - | - | T |
| 4 | *trn*T_L | 296 | i | T | - |  | T | T | - | - | - | T | - | T | T | T | T | - | T | T | - | - | T | T | T |
| 5 | *trn*T_L | 297 | i | T | - |  | T | T | T | T | T | T | T | T | T | T | T | T | T | T | T | T | T | T | T |
| 6 | *trn*T_L | 366 | m | (ACATA)2 | (ACATA) |  | - | - | - | - | A | - | A | - | - | - | - | A | A | - | - | - | - | - | - |
| 7 | *trn*T_L | 405 | i | TATT | - |  | T | T | T | T | T | T | T | T | T | T | T | T | T | - | T | T | T | T | T |
| 8 | *trn*T_L | 406 | s | A | G | - | G | G | G | G | G | G | G | G | G | G | G | G | G | - | G | G | G | G | G |
| 9 | *trn*T_L | 423 | m | (TTTATA)2 | (TTTATA) |  | T | T | T | T | T | T | T | T | T | T | T | T | T | T | T | T | T | T | T |
| 10 | *trn*T_L | 455 | s | G | A |  | G | G | G | G | G | G | G | G | G | G | G | G | G | G | G | G | G | G | G |
| 11 | *trn*T_L | 487 | s | G | T |  | T | T | T | T | T | T | G | T | T | T | T | G | T | T | T | T | T | T | T |
| 12 | *trn*T_L | 700 | s | T | A |  | A | A | A | A | T | A | T | A | A | A | A | T | A | A | A | A | A | A | A |
| 13 | *trn*T_L | 882 | s | G | A |  | A | A | A | A | A | A | A | A | A | A | A | A | A | A | A | A | A | A | A |
| 14 | *trn*T_L | 918 | s | G | A |  | A | A | A | A | A | A | A | A | A | A | A | A | A | A | A | A | A | A | A |
| 15 | *trn*T_L | 949 | s | A | C |  | C | C | C | C | A | C | A | C | C | C | C | A | C | C | C | C | C | C | C |
| 16 | *trn*T_L | 970 | i | - | A |  | A | A | A | A | A | A | A | A | A | A | - | A | A | A | A | - | A | A | A |
| 17 | *ndh*F*-rpl*32 | 1028 | i | - | C |  | C | C | C | C | C | C | C | C | C | C | C | C | C | C | C | C | C | C | C |
| 18 | *ndh*F*-rpl*32 | 1110 | i | - | A |  | A | A | A | A | A | A | A | A | A | A | A | A | A | A | A | A | A | A | A |
| 19 | *ndh*F*-rpl*32 | 1111 | s | C | A |  | A | A | A | A | A | A | A | A | A | A | A | A | A | A | A | A | A | A | A |
| 20 | *ndh*F*-rpl*32 | 1143 | i | - | A |  | A | A | A | A | A | A | A | A | A | A | A | A | A | A | A | A | A | A | A |
| 21 | *ndh*F*-rpl*32 | 1149 | m | - | A | T | - | - | - | - | **A** | - | **A** | - | - | - | - | **A** | - | - | - | - | - | - | - |
| 22 | *ndh*F*-rpl*32 | 1177 | i | A | - |  | - | - | - | - | - | - | - | - | - | - | - | - | - | - | - | - | - | - | - |
| 23 | *ndh*F*-rpl*32 | 1178 | i | - | A |  | A | A | A | A | A | A | A | A | A | - | A | A | A | A | A | A | A | - | A |
| 24 | *ndh*F*-rpl*32 | 1198 | m | - | TGATT |  | T | T | T | T | - | T | - | T | T | T | T | - | T | T | T | T | T | T | T |
| 25 | *ndh*F*-rpl*32 | 1260 | i | - | T |  | T | T | T | T | T | T | T | T | T | T | T | T | T | T | T | T | T | T | T |
| 26 | *ndh*F*-rpl*32 | 1286 | s | C | A |  | A | A | A | A | A | A | A | A | A | A | A | A | A | A | A | A | A | A | A |
| 27 | *ndh*F*-rpl*32 | 1397 | s | T | G |  | G | G | T | T | T | G | T | T | G | G | G | T | T | T | T | T | T | G | G |
| 28 | *ndh*F*-rpl*32 | 1526 | s | C | A |  | A | A | C | C | A | A | A | C | A | A | A | A | C | C | A | A | A | A | A |
| 29 | *ndh*F*-rpl*32 | 1530 | i | - | C |  | - | - | - | C | - | C | - | C | - | - | - | - | - | C | - | - | - | - | C |

**Table S1.** *Continued*.

| **Polymorphism** | **Region** | **Position** | **Type** | **Var1** | **Var2** | **Var3** | **Variant haplotypes codes** | | | | | | | | | | | | | | | | | | |
| --- | --- | --- | --- | --- | --- | --- | --- | --- | --- | --- | --- | --- | --- | --- | --- | --- | --- | --- | --- | --- | --- | --- | --- | --- | --- |
|  |  |  |  |  |  |  | **20** | **21** | **22** | **23** | **24** | **25** | **26** | **27** | **28** | **29** | **30** | **31** | **32** | **33** | **34** | **35** | **36** | **37** | **38** |
| 1 | *trn*T_L | 199 | i | T | - |  | T | T | T | T | T | T | T | T | T | T | T | T | T | T | T | T | T | T | T |
| 2 | *trn*T_L | 242 | i | G | - |  | - | - | - | - | G | - | - | - | - | - | - | - | - | G | - | G | - | - | G |
| 3 | *trn*T_L | 295 | i | T | - |  | - | - | T | T | T | T | - | - | - | - | - | T | - | - | - | - | - | - | - |
| 4 | *trn*T_L | 296 | i | T | - |  | T | T | T | T | T | T | - | T | - | - | - | T | - | - | - | - | - | - | - |
| 5 | *trn*T_L | 297 | i | T | - |  | T | T | T | T | T | T | - | T | T | T | T | T | - | - | - | - | - | - | - |
| 6 | *trn*T_L | 366 | m | (ACATA)2 | (ACATA) |  | - | - | - | A | - | - | - | A | A | A | A | A | - | A | A | A | A | A | A |
| 7 | *trn*T_L | 405 | i | TATT | - |  | T | T | T | T | T | T | T | T | T | T | T | T | T | T | T | T | T | T | T |
| 8 | *trn*T_L | 406 | s | A | G | - | G | G | G | G | G | G | G | G | G | G | G | G | G | A | A | A | A | A | T |
| 9 | *trn*T_L | 423 | m | (TTTATA)2 | (TTTATA) |  | T | T | T | T | T | T | T | T | T | T | T | T | T | - | - | - | - | - | A |
| 10 | *trn*T_L | 455 | s | G | A |  | G | G | G | G | G | G | G | G | G | G | G | G | G | A | A | A | A | A | G |
| 11 | *trn*T_L | 487 | s | G | T |  | T | T | T | T | T | T | T | G | G | G | T | T | T | T | T | T | T | T | T |
| 12 | *trn*T_L | 700 | s | T | A |  | A | A | A | A | A | A | A | T | T | T | T | A | A | A | A | A | A | A | A |
| 13 | *trn*T_L | 882 | s | G | A |  | A | A | A | A | A | A | A | A | A | A | A | G | A | A | A | A | A | A | A |
| 14 | *trn*T_L | 918 | s | G | A |  | A | A | A | A | A | A | A | A | A | A | A | A | G | G | G | G | G | G | G |
| 15 | *trn*T_L | 949 | s | A | C |  | C | C | C | C | C | C | C | A | A | A | C | C | C | A | A | A | A | C | A |
| 16 | *trn*T_L | 970 | i | - | A |  | A | A | A | A | A | A | A | A | A | A | A | A | A | A | A | - | A | - | A |
| 17 | *ndh*F*-rpl*32 | 1028 | i | - | C |  | C | - | C | C | C | C | C | C | C | C | C | C | C | C | C | C | C | C | C |
| 18 | *ndh*F*-rpl*32 | 1110 | i | - | A |  | A | A | A | A | A | - | A | A | A | A | A | A | A | A | A | A | A | A | A |
| 19 | *ndh*F*-rpl*32 | 1111 | s | C | A |  | A | A | A | A | A | A | A | A | A | A | C | A | A | A | A | A | A | A | A |
| 20 | *ndh*F*-rpl*32 | 1143 | i | - | A |  | - | A | A | A | A | A | A | A | A | A | A | A | A | A | A | A | A | A | A |
| 21 | *ndh*F*-rpl*32 | 1149 | m | - | A | T | - | - | - | - | - | - | - | **A** | - | **A** | **A** | **A** | **T** | **T** | **T** | - | - | **T** | **T** |
| 22 | *ndh*F*-rpl*32 | 1177 | i | A | - |  | - | - | - | - | - | - | - | - | A | - | - | - | - | A | A | A | A | A | A |
| 23 | *ndh*F*-rpl*32 | 1178 | i | - | A |  | A | A | A | A | A | A | A | A | A | A | A | - | A | A | A | A | A | A | A |
| 24 | *ndh*F*-rpl*32 | 1198 | m | - | TGATT |  | T | T | - | T | T | T | T | - | - | - | - | - | - | - | - | - | - | - | - |
| 25 | *ndh*F*-rpl*32 | 1260 | i | - | T |  | T | T | T | T | T | T | T | T | T | T | T | T | T | T | - | T | T | T | T |
| 26 | *ndh*F*-rpl*32 | 1286 | s | C | A |  | A | A | A | A | A | A | A | A | A | A | A | A | A | A | C | A | A | A | A |
| 27 | *ndh*F*-rpl*32 | 1397 | s | T | G |  | G | G | G | G | G | G | G | T | T | G | T | T | G | T | T | T | T | T | T |
| 28 | *ndh*F*-rpl*32 | 1526 | s | C | A |  | A | A | A | A | A | A | A | A | A | A | A | A | A | A | A | A | A | A | A |
| 29 | *ndh*F*-rpl*32 | 1530 | i | - | C |  | - | - | - | - | - | - | - | - | - | - | - | - | C | - | - | - | - | - | - |
